# Long-Term Responders After Autologous Stem Cell Transplantation in Multiple Myeloma

**DOI:** 10.3389/fonc.2022.936993

**Published:** 2022-07-05

**Authors:** Aina Oliver-Caldes, Juan Carlos Soler-Perromat, Ester Lozano, David Moreno, Alex Bataller, Pablo Mozas, Marta Garrote, Xavier Setoain, Juan Ignacio Aróstegui, Jordi Yagüe, Natalia Tovar, Raquel Jiménez, Luis Gerardo Rodríguez-Lobato, M. Teresa Cibeira, Laura Rosiñol, Joan Bladé, Manel Juan, Carlos Fernández de Larrea

**Affiliations:** ^1^ Hematology Department, Amyloidosis and Myeloma Unit, Hospital Clinic de Barcelona, Barcelona, Spain; ^2^ Institut d’Investigacions Biomèdiques August Pi i Sunyer (IDIBAPS), Barcelona, Spain; ^3^ Radiology Department Centre de Diagnòstic per la Imatge Clínic (CDIC), Hospital Clinic de Barcelona, Barcelona, Spain; ^4^ Department of Cell Biology, Physiology and Immunology, Faculty of Biology, Universitat de Barcelona (UB), and Institute of Biomedicine of the University of Barcelona (IBUB), Barcelona, Spain; ^5^ Hematopathology Unit, Pathology Department, Hospital Clinic de Barcelona, Barcelona, Spain; ^6^ Nuclear Medicine Department, Hospital Clinic de Barcelona, Biomedical Imaging Group, Biomedical Research Networking Center in Bioengineering, Biomaterials and Nanomedicine (CIBER-BBN), Barcelona, Spain; ^7^ Immunology Department, Hospital Clinic de Barcelona, Barcelona, Spain

**Keywords:** multiple myeloma, long-term responders, autologous stem cell transplantation, positron emission tomography/computed tomography, oligoclonal bands, T cell clones

## Abstract

**Introduction:**

Multiple myeloma (MM) is considered an incurable hematological neoplasm. For transplant-eligible patients, initial treatment includes an induction phase followed by an autologous stem cell transplantation (ASCT). Despite the introduction of several drugs in the past years, relapses still occur. Nevertheless, some patients achieve sustained responses after successful induction treatment and ASCT.

**Methods:**

We retrospectively evaluated all patients diagnosed with MM in our institution who underwent induction treatment and ASCT between 1990 and 2015. The subset of patients who achieved a sustained response (any degree) for 5 or more years after ASCT without further treatment or signs of progression were distinguished as “long-term responders” (LTRs). In the non-LTR group, a cohort referred to as “prolonged responders” (PLRs) showed sustained response of at least 5 years after ASCT but eventually relapsed. We collected and analyzed clinical and laboratory data.

**Results:**

Two hundred and fifty patients were diagnosed with MM and received induction treatment and ASCT at our institution in the study period. Among them, 54 (21.6%) patients met the criteria for LTR. Some diagnostic features such as a younger age, female gender, ECOG performance status of 0, lower International Staging System (ISS) stage, lower bone marrow plasma cell infiltration, and lower serum levels of calcium, C-reactive protein, and lactate dehydrogenase (LDH) were found to be more prevalent in LTR. Female gender, an ECOG performance status of 0, a localized Durie-Salmon stage, an ISS of I–II, the absence of bone disease, and an LDH within normal range were also predictive of longer progression-free survival (PFS) and overall survival (OS) in the whole cohort. The depth of the response achieved after induction and ASCT as well as the administration of an IMID-based maintenance regimen may play a role in the differences observed on PFS between cohorts. A detectable M-protein with a monoclonal gammopathy of undetermined significance (MGUS)-like behavior was detected in one-third of LTR after ASCT. Although relapses continue to occur in patients who achieve a 5-year treatment-free period after ASCT, a plateau is observed in the survival curves at approximately 21 years of follow-up.

## Introduction

The treatment of multiple myeloma (MM) has significantly evolved in the past decades, from polychemotherapy and the introduction of high-dose melphalan and autologous stem cell transplantation (ASCT), to the success of novel drugs such as proteasome inhibitors (PIs), immunomodulatory agents (IMIDs), monoclonal antibodies, and the recent advent of immunotherapy (1, 2). Despite these therapeutic improvements, MM remains virtually incurable, with a progressively shorter duration of response to each consecutive line of treatment (3, 4). However, some studies have reported a subset of ASCT-eligible patients who remain untreated for years after ASCT (5, 6), sometimes referred to as “operational cure”. Thus, a few questions arise regarding the chance of cure in these patients, as well as the possibility to detect predictive features that may help clinicians identify these patients.

Certain clinical and laboratory features at diagnosis have been reported to be more likely found in patients who did not require further treatment after ASCT such as a younger age, a lower ECOG (Eastern Cooperative Oncology Group) performance status, a higher hemoglobin and creatinine clearance, a lower ISS stage, and a lower number of high-risk cytogenetic abnormalities. Moreover, the achievement of complete response (CR) after ASCT has been associated with an improved survival in this disease (7–9).

After an induction therapy with novel drugs followed by ASCT, the option of adding some consolidation cycles and, particularly, maintenance therapy with low-dose anti-myeloma drugs arose, with the aim of lengthening time to relapse. A recent prospective, randomized, phase III clinical trial did not demonstrate an improvement of progression-free survival (PFS) or overall survival (OS) with the addition of consolidation therapy (10). However, maintenance with lenalidomide has been associated with significant PFS and OS improvement in several clinical trials for newly diagnosed MM patients (11, 12) and it is nowadays standard clinical practice worldwide.

In terms of bone disease, 18F-Fluorodeoxyglucose (FDG) positron emission tomography (PET) combined with computed tomography (CT) can accurately and sensitively detect MM bone lesions and extramedullary disease, estimating tumor metabolic activity and monitoring response to treatment (13). However, bone lytic lesions found in CT images alone are not always indicative of disease activity since residual lesions can frequently be found in treatment responders. Thus, the most important benefit of 18F-FDG PET/CT is its capability to accurately assess the burden of the disease and to differentiate between metabolically active and inactive bone lesions (14, 15).

Furthermore, some MM patients develop an oligoclonal humoral response after treatment with the emergence of a serum M-protein with an isotype that is different from the one found at diagnosis, called serum oligoclonal band (OB). Some studies have suggested that the presence of OB is associated with a favorable outcome (16, 17).

The aim of the present study is to describe the characteristics and outcome of newly diagnosed MM patients of a single institution who have not required further treatment after induction therapy and ASCT, and to compare them with the group of MM patients who experience relapse or refractoriness receiving the same therapy during the same period of time. We also evaluated the depth of response at different time points, the prognostic impact of lytic lesions after treatment, and the presence of OB and T cell clonality in LTR.

## Materials and Methods

### Patient Cohorts

In the present study, we included patients with newly diagnosed MM at our institution between 1990 and 2015 who were eligible for ASCT. Of these, we selected the patients who were alive and without further line of treatment for MM at least 5 years after ASCT, regardless of the response achieved. Consequently, patients who progressed and required treatment at any time during follow-up were discarded. The remaining patients were denominated long-term responders (LTRs). In the non-LTR group, some patients achieved a prolonged response (>5 years) but eventually relapsed. This subgroup was labeled prolonged responders (PLRs) and was considered for certain analyses. Patients who received consolidation or maintenance therapy according to local protocols were also analyzed.

Sample collection and clinical record review were performed after informed written consent in accordance with the Declaration of Helsinki. The study protocol was approved by the Institutional Review Board of Hospital Clinic of Barcelona. Patients were diagnosed according to standard International Myeloma Working Group (IMWG) criteria (18). Clinical and laboratory data were collected from medical reports in our institution, registered, and analyzed.

A subset of LTRs were referred for a 18F-FDG PET/CT radiologic evaluation of the bone disease status years after treatment. The median time between ASCT and PET/CT was 97 months. Baseline whole PET/CT images from skull to toe were acquired 60 min after intravenous administration of 3.7 MBq/kg of 18F-FDG, by means of hybrid PET/CT equipment (Biograph mCT TrueV, Siemens Medical Solutions USA, Inc.), including 8–10 beds (2 min per bed). All patients underwent low-dose CT for attenuation correction.

OBs were defined as previously described (16) as the presence of a serum and/or urine immunofixation (IF) monoclonal spike that was different from the original myeloma protein either in their heavy and/or light chains, as well as a different IF migration pattern.

### Flow Cytometry Analysis

T-cell clonal expansion was detected using TCR Vβ repertoire analysis by means of an IOTest Beta Mark TCR V Kit (Beckman Coulter, Brea, CA, USA). Relative frequencies of 24 Vβ T cell receptor families were analyzed in CD4+ and CD8+ T cells by flow cytometry in the peripheral blood of 17 recipients of ASCT from the LTR cohort. TCR Vβ panels included antibodies against CD3 (clone UCHT1), CD4 (clone 13B8.2), and CD8 (clone T8) (Beckman Coulter). Cells were acquired on a BD FACSCanto II cytometer and data were analyzed with FlowJo Software v.10 (BD Biosciences, San Jose, CA, USA).

### Statistical Analyses

Statistical analyses were performed with SPSS Statistics Data Editor v.25 (IBM Corp., Armonk, NY, USA). Chi-square or Fisher’s test were applied for categorical variables, and the Student’s *t*-test and Mann–Whitney *U* test were used for numerical variables when appropriate. *Post hoc* and planned comparison procedures for interpreting chi-square contingency table test results and the Bonferroni adjustment were also applied when appropriate (19). Survival was analyzed using Mantel–Cox and proportional hazard regression models, including pairwise comparisons. A *p*-value less than 0.05 was considered statistically significant.

## Results

### Clinical Features at Diagnosis

A total of 250 patients were diagnosed with MM and treated with ASCT as first-line treatment in our institution between 1990 and 2015. Of these, 11 patients (4.4%) died without relapse and 1 patient was lost to follow-up within the 5 years after ASCT; for this reason, these 12 patients were excluded from the analysis. The causes of death were infection (*n* = 8), toxicity (*n* = 2), and a cranial hemorrhage related to arterial hypertension (*n* = 1). Twenty-three patients achieved a prolonged response (>5 years) but eventually relapsed (PLR). From the remaining 238 patients, 54 (21.6% of *n* = 250) were found to meet the criteria of LTR and 184 were considered to be non-LTR.

The median follow-up after ASCT for the whole cohort was 78 months (range 4–321) and 141 months (61–321) for LTR. The mean time from diagnosis to ASCT was 8 months in the LTR cohort and 9 months in the non-LTR cohort.

The main characteristics of the patients at diagnosis in the LTR and non-LTR groups are shown in **Table 1**. A younger age, female sex, a lower International Staging System (ISS), an ECOG performance status of 0, a lower proportion of bone marrow (BM) plasma cells (PCs) by morphology in the BM aspirate, a lower C-reactive protein, lower calcium levels, and an LDH within normal range were more frequently found in the LTR cohort, compared with the non-LTR group (**Table 1**). The proportion of patients with a localized Durie-Salmon (DS) stage and the mean albumin levels were also higher in the LTR group, but not reaching statistical significance (DS *p* = 0.081; albumin *p* = 0.051). The same features were compared between the LTR cohort (*n* = 54) and the PLR cohort (*n* = 23) and the proportion of patients with an ECOG performance status of 0 was 50% vs. 9.1% (*p* = 0.01), respectively, being the only variable retaining statistical significance (**Table 1 Supplementary Material**).

**Table 1:** Main characteristics of long-term responders (LTR) vs. non-LTR.

Characteristics	Non-LTR (*n* = 184)	LTR (*n* = 54)	*p*-value
**Age at diagnosis** Median (range); years	55 (36–69)	52 (35–67)	**0.033**
**Sex** Female (%)/Male (%)	42/58	57/43	**0.043**
**Heavy chain isotype (**%) IgG IgA Bence Jones IgD Biclonal Non-secretory	59 23 16 1.6 0 1.1	65 20 11 1.9 1.9 0	NS
**Light chain isotype** κ %/λ (%) Biclonal (%)/Non-secretory (%)	63/37 0.5/0	61/37 0/1.9	NS
**Previous gammopathy**; (*n*); (%) MGUS SM Solitary plasmacytoma	62;34 35;19 23;13 4;2.2	20;37 9;17 6;11 5;9.5	NS
**Durie-Salmon stage** Localized (I–II) (%)/Advanced (III) (%)	50/50	63/37	NS
**ISS** I (%)/II (%)/III (%)	42/35/23	60/30/9.4	**0.034**
**ECOG** (%) 0 1–2 3–4	18 74 8.7	50 48 2.5	**<0.0001** NS NS
**Bone disease** (%) Osteolysis	71	52	**0.008**
**Extramedullary disease (**%)	26	32	NS
**Cytogenetic abnormalities (**% of pts)^1^ **Cytogenetic abnormalities** (*n*) t(11;14) IgH translocation (unknown partner) +1q Deletion Rb Other **High-risk cytogenetics** t(4;14) t(14;16) TP53 deletion	47 (*n* = 31/66)^2^ 11 1 1 4 10 4 0 6	44 (*n* = 11/25)^3^ 1 2 2 4 2 2 1 2	NS
**Serum M protein at diagnosis** Mean (g/L) Serum M protein > 30 g/L (% of pts)	36 60	30 47	NS NS
**Proteinuria** Mean (g/24 h)	1.8	1.3	NS
**BM plasma cells at diagnosis** Median (%)	47	32	**0.001**
**Creatinine** Mean (mg/dl)	1.50	1.25	NS
**Calcium** Mean (mg/dl)	9.9	9.4	**0.001^4^**
**Protein** Mean (g/L)	93.6	88.3	NS
**Albumin** Mean (g/L)	38.1	40	NS
**PCR** Mean (mg/dl)	1.96	0.48	**0.001**
**β2-microglobulin** Mean (mg/L)	4.6	3.7	NS
**High LDH** (%)	13	2.1	**0.036**
**Blood counts** Mean hemoglobin (g/L) Mean platelet count (×10^9^/mm^3^)	11.4 2.27	11.7 2.37	NS NS

Numbers > 10 were rounded to the closest whole number in categorical variables.BM, bone marrow; ECOG, Eastern Cooperative Oncology Group; ISS, International Staging System; MGUS, monoclonal gammopathy of undetermined significance; LDH, lactate dehydrogenase; NS, non-statistically significant; PCR, C-reactive protein; Pts, patients; SM, smoldering myeloma. ^1^Data from *n* = 91 patients. ^2^We observed 5 patients with 2 or more cytogenetic abnormalities with a total of *n* = 37 cytogenetic abnormalities. ^3^We observed 5 patients with 2 cytogenetic abnormalities. ^4^The difference is not clinically significant. The bold values refer to the ones that al statistically significant, which would be the values <0.05.

### Differences in Response to Treatment

**Figure 1:**
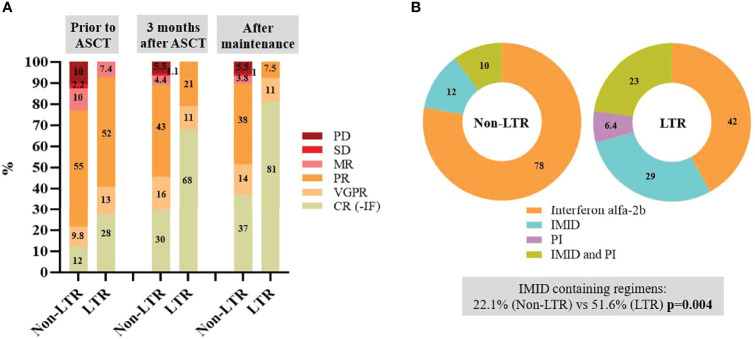
**(A)** Responses obtained prior to autologous stem cell transplantation (ASCT), after 3 months of ASCT and global best response obtained after ASCT with or without consolidation and maintenance therapy in long-term responders (LTR) and non-LTR. **(B)** Proportion of patients receiving each treatment-based maintenance in LTR and non-LTR. All percentages were rounded to the closest absolute number when > 10. CR, complete response; IF, immunofixation; IMID, immunomodulatory drugs; MR, minimal response; PD, progressive disease; PI, proteasome inhibitors; PR, partial response; SD, stable disease; VGPR, very good partial response.

The responses achieved prior to ASCT in LTR vs. non-LTR were as follows: complete response (CR) 27.8% vs. 12%, very good partial response (VGPR) 13% vs. 9.8%, partial response (PR) 51.9% vs. 55.2%, minimal response (MR) 7.4% vs. 10.4%, stable disease (SD) 0 vs. 2.2%, and progressive disease (PD) 0 vs. 10.4% (**Figure 1A**). The achievement of any degree of response to the first line of induction treatment was higher in the LTR group [90.7% vs. 72.8% (*p* = 0.006)]. Of note, a bortezomib-containing regimen was received by 55% LTR vs. 29% non-LTR (*p* < 0.001) and a lenalidomide-containing regimen was received by 21% LTR vs. 3% non-LTR (*p* < 0.001). None of the patients received anti-CD38 antibody, such as daratumumab, in first-line treatment for historical reasons.

The responses achieved at 3 months from ASCT in LTR vs. non-LTR were CR with negative IF in 67.9% vs. 29.7% (*p* < 0.001), VGPR 11.4% vs. 15.9%, PR 20.8% vs. 43.4% (*p* = 0.0027), MR 0 vs. 4.4%, SD 0 vs. 1.1%, and PD 0 vs. 5.5% (**Figure 1A**).

In this cohort of 238 patients, due to the period of time in which they were diagnosed and treated, only 5.9% received consolidation treatment (LTR 18.5% vs. non-LTR 2.2%; *p* < 0.001). All patients received a bortezomib-, lenalidomide-, and dexamethasone-based scheme of consolidation. Maintenance was administered to 38.6% of patients (LTR 57.4% vs. non-LTR 33%; *p* = 0.001). From the patients who received maintenance in each group, an interferon alpha-2b-based scheme was administered to 42% LTR vs. 78% non-LTR; IMID-based in 29% (LTR) vs. 11.9% (non-LTR), PI-based in 6.4% (LTR) vs. 0 (non-LTR), and a combination of PI and IMID in 22.6% (LTR) vs. 10.2% (non-LTR) (**Figure 1B**). The main reason for ending maintenance therapy in the non-LTR group was disease progression (58%) followed by drug toxicity (33%), while end of treatment by protocol (59%) was the main reason in the LTR group, also followed by drug toxicity (30%).

In terms of global response between LTR vs. non-LTR after induction therapy, ASCT, and consolidation/maintenance, if received, a CR was observed in 81.1% vs. 37.2% (*p* < 0.001), VGPR 11.4% vs. 14.2%, PR 7.5% vs. 38.3% (*p* < 0.001), MR 0 vs. 3.8%, SD 0 vs. 1%, and PD 0 vs. 5.5% (**Figure 1A**). Of note, 18.9% of LTR did not achieve a CR with negative IF. Thus, as much as one-third (*n* = 18) of LTR had a detectable serum monoclonal (M) protein of the same isotype than at diagnosis over the evolution of the disease, either as a result of a PR or VGPR after maintenance or due to a serological relapse after achieving response. None of these patients developed CRAB symptoms or required treatment during follow-up.

When comparing the LTR and the PLR cohorts, no statistically significant differences were seen in terms of response prior to ASCT, at 3 months from ASCT or after maintenance, although there was a trend towards deeper responses in the LTR cohort (**Figure 1A Supplementary Material**). Maintenance was administered in 57% (LTR) vs. 59% (PLR), with no significant differences in the regimens used (**Figure 1B Supplementary Material**).

### Survival Analysis

**Figure 2:**
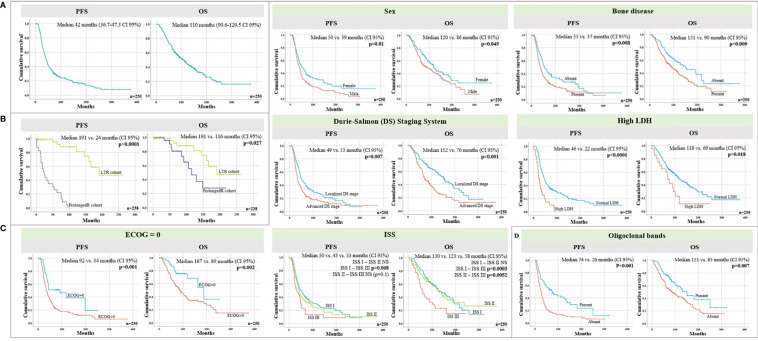
**(A)** Progression-free (PFS) and overall survival (OS) of the complete cohort of *n* = 250 patients. **(B)** Progression-free (PFS) and overall survival (OS) of the prolonged response (PLR) cohort (dark blue) and long-term responders (LTR) cohort (green). Time 0 refers to a landmark time set at 5 years after transplant. **(C)** PFS and OS in the whole cohort (*n* = 250) according to different variables at the time of diagnosis: Eastern Cooperative Oncology Group (ECOG) = 0 vs. ECOG > 0 performance status, normal vs. high dehydrogenase lactate (LDH), sex, localized vs. advanced Durie-Salmon (DS) stage, International Staging System (ISS), and the presence of bone disease. **(D)** PFS and OS from ASCT according to the presence or absence of oligoclonal bands at response evaluation. DS Stages I and II were considered localized DS stage and DS stage III was considered advanced stage.

The median PFS for the whole cohort of 250 patients (including patients who died without relapse within 5 years after ASCT) was 42 months (95% CI: 36.7–47.3), with a median OS of 110 months (95% CI: 90.6–129.5) (**Figure 2A**). A landmark analysis was performed to determine median PFS and OS of the PLR cohort vs. the LTR cohort, considering time 0 as a landmark time set at 5 years after ASCT (**Figure 2B**). As expected, median PFS was 191 (LTR) vs. 24 (PLR) months (*p* < 0.0001), due to the definition of each cohort. The 9 events observed in the LTR cohort corresponded to 9 deaths due to the following causes: 4 second primary malignancies (breast, colon, therapy-related acute myeloid leukemia, and unknown origin), 1 sepsis, 1 meningitis, 1 traumatic brain injury, 1 ischemic stroke, and 1 suspected wild-type transthyretin cardiomyopathy while remaining in CR of the MM. Median OS was 191 (LTR) vs. 116 (PLR) months (*p* = 0.027).

Some baseline characteristics of MM were found to be predictive of survival in the whole cohort (*n* = 250) of patients (**Table 2**; **Figure 2C**). In the group of patients who remained untreated 5 years after ASCT, which included the LTR and the PLR cohorts, the landmark analysis showed that the median PFS in female patients was longer, with a trend towards statistical significance (*p* = 0.063), although no differences were found in terms of OS (**Figure 2 Supplementary Material**).

**Table 2:** Survival data according to different characteristics at diagnosis.

	PFS		OS	
	Median (months) or Hazard ratio (95% CI)	*p*-value	Median (months) or Hazard ratio (95% CI)	*p*-value
**Sex** Female vs. male	50 vs. 39	0.01	120 vs. 86	0.049
**ECOG performance status scale** =0 vs. >0	92 vs. 34	0.001	187 vs. 89	0.002
**Durie-Salmon Staging System** Localized vs. advanced	49 vs. 33	0.007	152 vs. 70	<0.001
**ISS** I vs. II vs. III I vs. II I vs. III II vs. III	50 vs. 43 vs. 33	NS 0.008 NS	130 vs. 123 vs. 58	NS 0.0003 0.0052
**Bone disease** Absent vs. present	55 vs. 37	0.008	151 vs. 90	0.009
**LDH** Normal vs. elevated	46 vs. 22	<0.0001	118 vs. 69	0.018
**BM plasma cells** %	1.007	0.028	1.008	0.036
**Serum calcium levels** mg/dl	1.232	0.018	1.256	0.019

BM, bone marrow; ECOG, Eastern Cooperative Oncology Group; ISS, International Staging System; LDH, lactate dehydrogenase; NS, non-statistically significant; PFS, progression-free survival; Pts, patients; OS, overall survival.

### Imaging Analysis With 18F-FDG PET/CT

**Figure 3:**
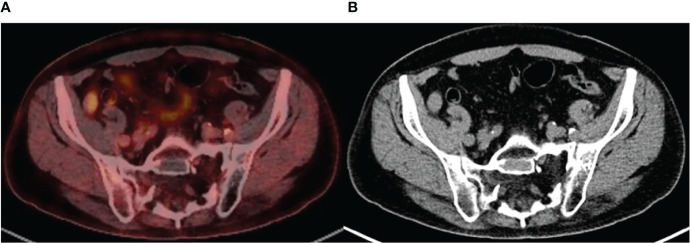
**(A)** Axial fused positron emission tomography/computed tomography (PET/CT) image demonstrates a large sacral lytic bone lesion, with no significant fluorodeoxyglucose (FDG) uptake. Findings are consistent with inactive lesion. **(B)** Axial CT image shows characteristics of residual bone lesion: sclerotic margins and fat content. Note that the few oval dense spots inside the lesion are the sacral roots passing through the lytic area. Findings are consistent with a chronic residual lesion.

In our cohort of patients, 16 out of 54 LTRs were referred for 18F-FDG PET/CT. Thirteen out of 16 (81%) LTRs with 18F-FDG PET/CT showed lytic bone lesions in CT images. None of these lesions showed FDG uptake in the PET images, so they were classified as inactive lesions. In these patients, CT images showed patterns that are characteristic of residual chronic lesions, such as sclerotic bone margins and fat replacement of the lesion. CT images from MM patients will continue to show these residual lesions for a long period, but they can be classified as inactive lesions when there is no significant FDG uptake in PET images (**Figure 3**).

### Presence of Oligoclonal Bands and T Cell Clonality in LTR

**Figure 4:**
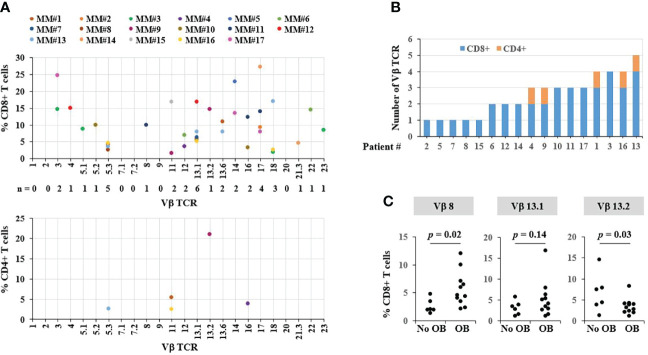
Analysis of T-cell clonality in long-term responders (LTR). **(A)** Frequencies of CD8+ and CD4+ T cells expressing each Vβ TCR subset measured by flow cytometry. Each dot represents one measurement; only values greater than normal values are depicted. **(B)** Number of Vβ TCR per patient. **(C)** TCR Vβ subsets in CD8+ T cells from patients with and without oligoclonal bands (OB).

We then evaluated whether T-cell clonal expansion could also be involved in the achievement of a sustained remission. For that purpose, relative frequencies of 24 Vβ T cell receptor (TCR) families were analyzed in CD4^+^ and CD8^+^ T cells by flow cytometry in peripheral blood samples of 17 out of the 54 patients in the LTR group. As expected, the Vβ TCR repertoire in CD8^+^ T cells showed higher diversity in the number of over-represented TCR Vβ compared to CD4^+^ T cells (**Figure 4A**). All the patients studied showed at least one TCR Vβ overrepresented in CD8^+^ T cells (**Figure 4B**), suggesting an enrichment compared to the general population.

In the LTR group, 71.2% of patients presented OB in the 3-month follow-up after ASCT, compared to 28.7% in the non-LTR (*p* < 0.0001). When considering separately the 3 cohorts, OBs were observed in 71.2% of LTR, 47.6% of PLR, and 26% of non-LTR (PLR vs. non-LTR *p* = 0.041; PLR vs. LTR *p* = 0.057). OBs were associated with a longer PFS (*p* < 0.001) and OS (0.007) in the cohort of patients with response evaluation after ASCT, and no differences were observed in the landmark analysis performed at 5 years from ASCT including LTR and PLR. Accordingly, in the cohort selected for TCR analysis, 11 out of 17 patients (65%) showed an OB in the serum immunofixation. Among these patients with an oligoclonal humoral immune response, the most frequent overrepresented TCRs were Vβ 8 and 13.1 (**Figure 4C**). In contrast, TCR Vβ 13.2 was higher in patients who did not show OBs. Taken together, our data showed that T-cell clonality may play a role in achieving sustained responses after ASCT.

## Discussion

In this study, 22% of patients treated with induction therapy and ASCT after MM diagnosis in a single institution were found to meet the criteria for LTR. Several features were more prevalent in the LTR group, compared to the non-LTR. In terms of survival, an ECOG performance status of 0, a less advanced stage (by DS stage or ISS of I–II), the absence of bone disease, and an LDH within normal range were predictive of longer PFS and OS in the whole cohort of 250 patients. While a younger age and a normal ECOG performance status seem to be related to a better general condition of the patient and a higher probability of undergoing ASCT, both a lower DS stage and a lower ISS stage, the absence of bone disease, a lower proportion of BM PC, and lower levels of calcium, C-reactive protein, and LDH may be ultimately related to a less aggressive disease.

Interestingly, in this cohort, a higher proportion of LTR were women, with sex being predictive of a longer PFS and OS. Previous studies have not reported statistically significant differences regarding sex in MM patients who achieve prolonged PFS, although study designs were not absolutely comparable (5, 20).

High-risk (HR) cytogenetic abnormalities were found in 20% (LTR) vs. 15% (non-LTR) of the patients with available cytogenetic data [HR was established according to the IMWG definition (21)]. This is inconsistent with the reported data that suggest that HR abnormalities confer a worse prognosis, with more aggressive disease, lower response to conventional treatment, and shorter duration (22). However, this study is limited by the fact that the cytogenetic data of this cohort were evaluable only in a fraction of patients (38%), and that many of the results were obtained from conventional cytogenetics and not CD138-isolated cells using fluorescence *in situ* hybridization (FISH) testing. However, it is true that some LTRs are carrying out HR cytogenetic risk, confirming the heterogeneity of the prognosis in patients with HR in some series, with some of them surviving as standard cytogenetic risk. The exact proportion of these patients and the long-term impact of each cytogenetic abnormality to achieve LTR status should be explored prospectively.

The depth of the response achieved prior to ASCT, after ASCT, and after maintenance, when administered, was significantly higher in the LTR group, which is consistent with previous observations (23). In this study, only a minority of patients had a high-sensitivity minimal residual disease evaluation, since it was not systematically performed throughout the study period. Although the majority of patients in the LTR group achieved a CR with negative IF after ASCT or consolidation/maintenance therapy, 19% remained IF-positive or presented some amount of M protein in the serum. Also, some low-grade relapses with reappearance of the serum M protein were observed in the LTR group, with no symptoms or need for further treatment. This is consistent with the previous observations reported by several studies (9, 20, 24), in line with the idea that, after treatment, some patients may have a relapse with an MGUS-like behavior. Even in the PLR cohort, relapses were reported up to 96 months (8 years) after ASCT; a plateau is observed in the PFS and OS curves of the whole cohort after 21 years of follow-up, suggesting a potential cure for a proportion of patients with MM.

Due to the period in which this cohort of patients was diagnosed and treated, medical management was not homogeneous over time. As mentioned before, a PI- and IMID-based induction regimen was more frequently administered to the LTR cohort. Moreover, consolidation and maintenance were more frequently administered in the LTR group. Also, a higher proportion of non-LTR received interferon alfa-2b as maintenance therapy, while single PIs, IMIDs, or a combination of both were more frequently received as maintenance therapy by LTR, although no statistically significant differences were observed. The differences in the management of these patients together with the favorable results reported with lenalidomide maintenance may be responsible for the better outcomes in LTR.

No significantly different features were observed between LTR and PLR but ECOG performance status at diagnosis, and no differences were observed in terms of depth of response prior to or after ASCT. Also, the percentage of patients receiving maintenance therapy was similar in both groups. Even though no statistical differences were detected, the regimen used for maintenance was slightly different, with more interferon alfa-2b-based therapy and less IMID-based therapy in the PLR cohort; this may have played a role in the lower duration of response in this group.

18F-FDG PET/CT imaging combines whole-body functional imaging with PET and morphologic imaging with CT in a single study (25). The role of 18F-FDG PET/CT in MM has achieved a highly significant level of evidence (15), being included in the International Myeloma Working Group (IMWG) (26) recommendations.

Bartel et al. stated that the number of PET-positive focal lesions at staging, the presence of PET-positive extramedullary disease at staging, and the suppression of FDG uptake after induction are associated with overall and event-free survival (27, 28). Zamagni et al. concluded that PET/CT involvement at diagnosis and after novel agent-based induction and subsequent ASCT is a reliable predictor of prognosis (29). However, the recommendation to perform an 18F-FDG PET/CT after ASCT is not as clear-cut in clinical practice. The interpretation of post-induction PET data is challenging to hematologists (27). A study by Hillner et al. (30) showed that among 18 different types of malignancies, 18F-FDG PET/CT had the highest effect on the management of patients with MM, resulting in a change in the treatment strategy in 49% of cases.

18F-FDG PET/CT discerns vital from non-vital osteolytic bone lesions, differentiating active myeloma from inactive lesions. Osteolytic lesions in MM hardly heal completely, even after many years, as we can observe in our series. In comparison, the role of CT and MRI after bone marrow transplant is more limited because non-vital osteolytic bone lesions may persist for years in patients who have achieved a CR (25), although the presence of fat replacement within the lesion and the appearance of sclerotic margins in CT images are strongly suggestive of inactivity.

In the present study, we observed a better prognosis associated to the emergence of OB. Molecular studies have demonstrated a non-clonal related origin of plasma cells in MM patients in complete remission with OB; it can result from a higher tumor reduction with a stronger immune reconstitution. We also observed that T-cell clonal expansion may be involved in the achievement of sustained remission. Characterization of the TCR repertoire in LTR revealed a higher involvement of the CD8^+^ T cells compared to CD4^+^ T cells, which is consistent with immunological studies in myeloma patients achieving long-term disease control showing increased frequencies of CD8^+^ T cells (31). This demonstrates that oligoclonal expanded CD8^+^ T cells are key to disease control in myeloma (32). Indeed, all the patients studied (*n* = 17/17) showed at least one overrepresented TCR Vβ in CD8^+^ T cells. Accordingly, Bryant et al. reported that 100% (*n* = 19/19) of the long-term-survivor (LTS) MM patients showed at least one TCR Vβ over-represented in CD3^+^ T cells (33), including Vβ 13.1, which we found increased in patients with oligoclonal humoral immune response. Taken together, our data showed that T-cell clonality may play a role in achieving a sustained remission.

In conclusion, the data reported from this cohort of patients with MM highlight that almost 22% of patients treated with induction and ASCT can achieve the LTR status; some characteristics at diagnosis may be predictive of better outcomes and PI- or IMID-based schemes of induction plus IMID-based maintenance regimens may play a role in the differences observed. Also, some LTRs have a detectable M-protein with no need for MM treatment, with an MGUS-like behavior that should be recognized in order to avoid overtreatment. Finally, even though relapses have been detected in patients with prolonged responses up to 8 years from ASCT, a plateau is observed in the survival curves.

## Data Availability Statement

The raw data supporting the conclusions of this article will be made available by the authors, without undue reservation.

## Ethics Statement

The studies involving human participants were reviewed and approved by Comitè d’Ètica d’Investigació (CEIm), Hospital Clinic de Barcelona. Written informed consent for participation was not required for this study in accordance with the national legislation and the institutional requirements.

## Author Contributions

AO-C, JS-P, XS, LR, JB, and CL participated in conception and design of the work. AO-C, JS-P, EL, and DM participated in the writing of the manuscript. AO-C, JS-P, EL, AB, PM, and XS provided data and figures. NT, RJ, LR-L, and MC collaborated with data collection. AO-C, DM, AB, PM, and CF performed data analysis and interpretation. MC, LR, JB, and CL revised the article. All the authors gave final approval of the version to be published.

## Funding

This work has been supported in part by grants from the Instituto de Salud Carlos III, Spanish Ministry of Health (FIS PI18/00775, PI19/00669, ICI19/00025, and a complementary grant for CONCORD-023; co-funded by the European Union) as well as from Asociación Española Contra el Cancer (AECC) LABAE21971FERN. AOC received funding from the resident award “Ajut Clínic-La Pedrera” 2019 (Hospital Clínic de Barcelona).

## Conflict of Interest

The authors declare that the research was conducted in the absence of any commercial or financial relationships that could be construed as a potential conflict of interest.

## Publisher’s Note

All claims expressed in this article are solely those of the authors and do not necessarily represent those of their affiliated organizations, or those of the publisher, the editors and the reviewers. Any product that may be evaluated in this article, or claim that may be made by its manufacturer, is not guaranteed or endorsed by the publisher.

## Abbreviations

ASCT, autologous stem cell transplantation; BM, bone marrow; CI, confidence interval; CR, complete response; CT, computed tomography; DS, Durie-Salmon; ECOG, Eastern Cooperative Oncology Group; FDG, fluorodeoxyglucose; FISH, fluorescence *in situ* hybridization; HR, high risk; IF, immunofixation; IMIDs, immunomodulatory agents; IMWG, International Myeloma Working Group; IFN, interferon alpha-2b; ISS, International Staging System; LDH, lactate dehydrogenase; LTR, long-term responder; M, monoclonal; MGUS, monoclonal gammopathy of undetermined significance; MM, multiple myeloma; MR, minimal response; OB, oligoclonal band; OS, overall survival; PC, plasma cell; PD, progressive disease; PET, positron emission tomography; PFS, progression-free survival; PIs, proteasome inhibitors; PR, partial response; PLR, prolonged responder; SD, stable disease; VGPR, very good partial response.
