# Supplementary material for: Long-Term Responders After Autologous Stem Cell Transplantation in Multiple Myeloma
Source: Front Oncol. 2022 Jul 5;12:936993. doi: 10.3389/fonc.2022.936993 (PMC9294166; doi:10.3389/fonc.2022.936993)
Supplement: Supplementary file 1 [file DataSheet_1.docx]

Supplementary Material

# Supplementary Figures and Tables

## Supplementary Figures

**
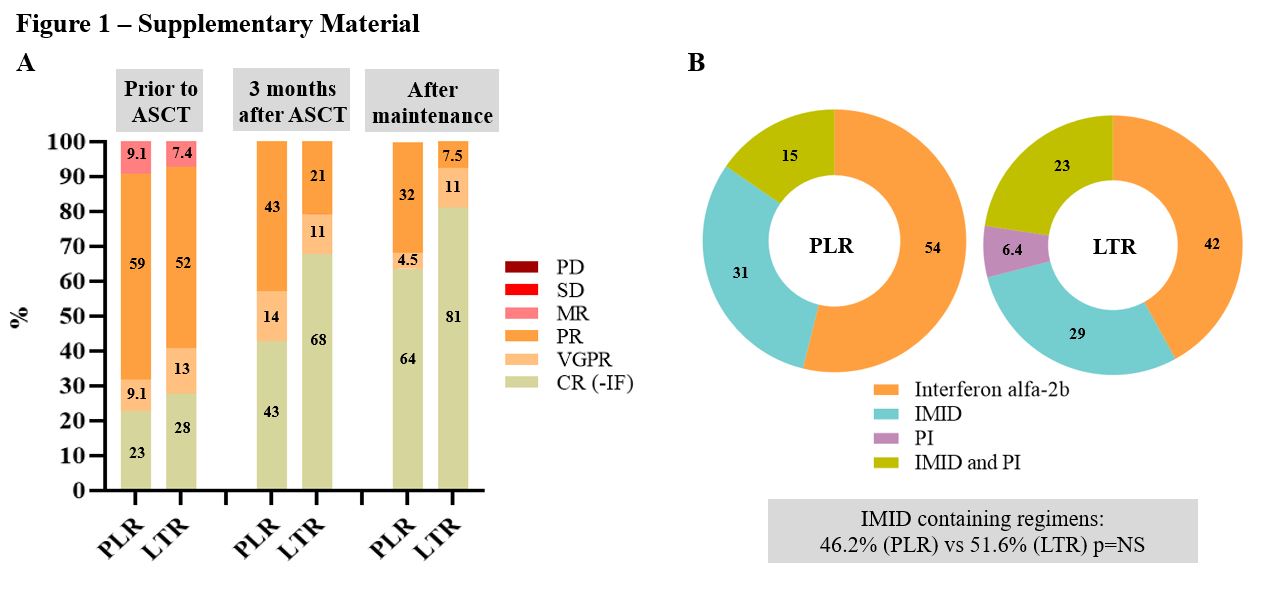
**

**Figure 1 – Supplementary Material.** (**A**) Responses obtained prior to autologous stem cell transplantation (ASCT), after 3 months of ASCT and global responses obtained after ASCT with or without consolidation and maintenance therapy in long-term Responders (LTR) vs. prolonged responders (PLR). (**B**) Proportion of patients receiving each treatment-based maintenance in LTR and PLR. All percentages were rounded to the closest absolute number when > 10. CR: complete response; IF: immunofixation; IMID: immunomodulatory drugs; MR: minimal response; PD: progressive disease; PI: proteasome inhibitors; PR: partial response; SD: stable disease; VGPR: very good partial response.

**
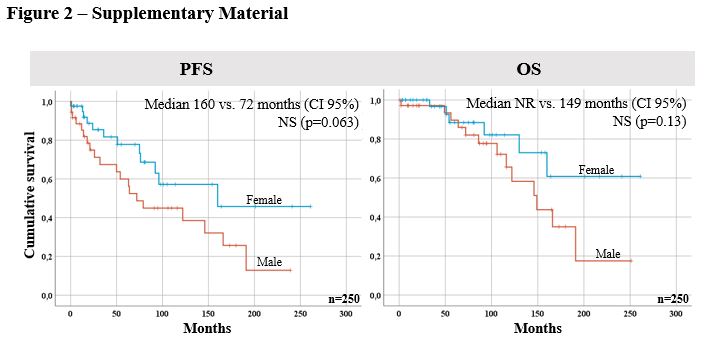
**

**Figure 2 - Supplementary Material.** Landmark analysis of progression-free (PFS) and overall survival (OS) according to sex in the group of patients remaining untreated 5 years after autologous stem cell transplantation (including long-term Responders (LTR) and prolonged Responders (PLR)). Time 0 was set at 5 years after autologous stem cell transplantation. NR: not reached; NS: non statistically significant.

## Supplementary Tables

**Table 1 – Supplementary Material.** Main characteristics of long-term Responders (LTR) vs. prolonged responders (PRL).

| **Characteristics** | **PLR (n=23)** | **LTR (n=54)** | **p-value** |
| --- | --- | --- | --- |
| **Age at diagnosis**  Median (range);years | 53 (36-67) | 52 (35-67) | NS |
| **Sex**  Female (%)/Male (%) | 44/56 | 57/43 | NS |
| **Heavy chain isotype** (%)  IgG  IgA  Bence-jones  IgD  Biclonal  Non-secretory | 70  8.7  22  1.6  0  0 | 65  20  11  1.9  1.9  0 | NS |
| **Light chain isotype**  κ (%)/λ (%)  Biclonal (%)/Non-secretory (%) | 70/30  0/0 | 61/37  0/1.9 | NS |
| **Previous gammopathy** (n); (%)  MGUS  SM  Solitary plasmacytoma | 48;34  5;22  6;26  0;2.2 | 20;37  9;17  6;11  5;9.5 | NS |
| **Durie-Salmon stage**  Localized (I-II) (%)/Advanced (III) (%) | 70/30 | 63/37 | NS |
| **ISS**  I (%) / II (%) / III (%) | 48/43/9.5 | 60/30/9.4 | NS |
| **ECOG** (%)  0  1-2  3-4 | 9.1  64  27 | 50  38  12 | **0.01**  NS  NS |
| **Bone disease** (%)  Osteolysis | 71 | 52 | NS |
| **Cytogenetic abnormalities** (% of pts)^1^  **Cytogenetic abnormalities** (n)  t(11;14)  IgH translocation (unknown partner)  +1q  Deletion Rb  Other  **High-risk cytogenetics** (n)  t(4;14)  t(14;16)  TP53 deletion | 20 (n=2/10)^2^  1  0  1  0  1  0  0  0 | 44 (n=11/25)^3^  1  2  2  4  2  2  1  2 | NS |
| **Serum M protein at diagnosis**  Mean g/dL  Serum M protein > 30 g/L (% of pts) | 34  57 | 30  47 | NS  NS |
| **Proteinuria**  Mean g/24 hours | 1.6 | 1.3 | NS |
| **BM plasma cells at diagnosis**  Median (%) | 43 | 32 | NS |
| **Creatinine**  Mean mg/dL | 1.05 | 1.25 | NS |
| **Calcium**  Mean mg/dL | 9.7 | 9.4 | NS |
| **Protein**  Mean mg/dL | 90.2 | 88.3 | NS |
| **Albumin**  Mean g/L | 39.3 | 40 | NS |
| **PCR**  Mean g/L | 0.97 | 0.48 | NS |
| **β2-microglobulin**  Mean mg/L | 3.2 | 3.7 | NS |
| **High LDH** (%) | 4.8 | 2.1 | NS |
| **Blood counts**  Mean hemoglobin (g/L)  Mean platelet count (x10^9^/mm^3^) | 11.4  2.26 | 11.7  2.37 | NS  NS |

Numbers > 10 were rounded to the closest whole number in categorical variables.

BM: bone marrow; ISS: international staging system; MGUS: monoclonal gammopathy of undetermined significance; LDH: lactate dehydrogenase; NS: non statistically significant; PCR: reactive C-protein; Pts: patients; SM: smoldering myeloma;

^1^Data of n=91 patients.

^2^We observed 1 patient with 2 cytogenetic abnormalities.

^3^We observed 5 patients with 2 cytogenetic abnormalities.
